# Predictors of HIV Knowledge, Perceived Stigma and Risk among Transport Workers in Mbarara City, Southwestern Uganda

**DOI:** 10.24248/eahrj.v8i2.787

**Published:** 2024-06-26

**Authors:** Benjamin Betunga, Lilian Nuwabine, Eve Katushabe, Grace Among, Mary Grace Nakate, Ahmed M Sarki, Diana Mbatudde, Mary Namuguzi, John Baptist Asiimwe

**Affiliations:** a Aga Khan University, Uganda Campus, Kampala, Uganda; b Faculty of Nursing and Health sciences, Bishop Stuart University, Mbarara, Uganda; c Family and Youth Health Initiative (FAYOHI), Dutse, Jigawa State, Nigeria

## Abstract

**Background:**

The human immunodeficiency virus (HIV) prevalence among transport workers in sub-Saharan Africa remains high, estimated at as high as 9.9% in western Uganda compared with the national prevalence of 5.4%. The prevalence of HIV among transport workers has been partly attributed to the level of knowledge regarding HIV prevention, perceived HIV risk, and stigma. Accordingly, these have been linked to high-risk HIV transmission behaviours that increase the chances of acquiring HIV among adults. Therefore, this study investigated the predictors of HIV knowledge, perceived HIV risk, and stigma among transport workers in Mbarara city in southwestern Uganda.

**Methods:**

The survey was conducted between November 2021 and February 2022 among transport workers (motorcycle taxi riders, motor vehicles taxi, and truck drivers), aged 18 to 55 years. Face-to-face interviews using a semi-structured questionnaire were conducted with the study's participants. Chi-square and binary multivariate logistic regression statistics were used to assess the predictors of knowledge about HIV prevention, HIV perceived risk, and stigma.

**Results:**

Out of 420 participants, 69.3%, 75.4%, and 62% had good knowledge of HIV prevention, a high perceived HIV risk, and stigma, respectively. Predictors of knowledge of HIV prevention comprised education level (AOR=2.28, 95% CI=1.36-3.84), knowing HIV status (AOR=0.47, 95% CI=0.27-0.81), and perceived HIV risk (AOR=3.04, 95% CI=1.74-5.32). Whereas the determinants of perceived HIV risk included education level (AOR=1.34, 95% CI=1.34-4.24), knowing HIV status (AOR=0.26, 95% CI=0.15-0.48), HIV knowledge (AOR=2.38, 95% CI=1.36-4.178), and perceived stigma (AOR=0.47, 95% CI=0.24-0.89). Last, the predictors of perceived HIV stigma included perceived HIV risk (AOR=0.41, 95% CI=0.21-.791), and knowledge of HIV prevention (AOR=0.29, 95% CI=0.16-0.54).

**Conclusions:**

The study found a high proportion of participants with good knowledge about HIV prevention, a high HIV perceived risk, and HIV-related stigma. In addition, this study suggests that the level of education and awareness of one's HIV status positively influences HIV knowledge and perceived risk. Whereas HIV-related stigma was in turn negatively influenced by the transport workers' HIV knowledge and perceived risk. This calls for multifaceted approaches at individual, group (interpersonal), and community levels to reduce HIV stigma among this study group. Incorporating continuous health education programs about HIV and encouraging HIV testing among transport workers remains critical.

## BACKGROUND

Over the past decades, there has been an increase in the availability and access to HIV prevention measures among adults. However, new HIV infections have not significantly abated globally.^1^ Individuals at risk of HIV infection, such as; men who have sex with men (MSM), those who inject drugs, sex workers, and their clients (predominantly transport workers), remain the key drivers of the HIV epidemic.^1,2^ The long stay away from their homes and families, transport workers, particularly truck drivers, are predisposed to transactional sex with sex workers or MSM along their transit routes, predisposing them to HIV infections. Studies have indicated that transport workers, most especially truck drivers, constitute 70% of the clients of sex workers.^1,3^ Additionally, transport workers contributed up to 26% of new HIV infections in 2020 in sub-Saharan Africa.^1,3,4^

In sub-Saharan Africa, HIV prevalence among transport workers exceeded the national prevalence statistics in some countries.^5^ For example, HIV prevalence among truck drivers in South Africa was reported at 26% compared to the national prevalence among adults of 18.3%.^6^ In Uganda, the prevalence of HIV among motorcycle taxi riders was reported at 9.9% in western Uganda compared to the national prevalence of 5.4% among adults aged 15 to 49 years.^7,8^

From a theoretical perspective, low levels of knowledge of HIV prevention, perceived HIV risk, and HIV stigma have been linked to high-risk HIV transmission behaviours that increase the chances of acquiring HIV among adults, particularly among key populations.^9-12^ Specifically, community-based studies indicate that HIV-related stigma remains high and is a global problem that negatively impacts HIV infection control by reducing the number of people seeking an HIV test, delaying linkage to HIV care for those with a positive HIV diagnosis, and reducing adherence to HIV drugs for those already enrolled into HIV care.^9,10^ Perceived HIV risk has also been documented as a key predictor of risky behaviours. This is because how one thinks and feels about their HIV risk, determines whether they engage in risky sexual behaviours that increase HIV transmission (such as having multiple concurrent sex partners) or non-risky behaviours such as the use of HIV prevention services that reduce the risk of HIV.^11^ Similarly, low knowledge about HIV transmission and prevention is associated with high-risk HIV transmission behaviours and lower intent to use HIV prevention measures such as condoms.^12^

Nonetheless, there are exceptions. Accordingly, the need to satisfy socio-economic needs, excellent knowledge about HIV transmission, and high perceived HIV risk were reported in individuals engaged in risky sexual behaviours. ^4^ Therefore, there have been suggestions that tackling low HIV knowledge or perceived HIV risk alone may not sufficiently reduce the risky sexual behaviours that perpetuate HIV transmission.^4^

Nevertheless, factors that predict the level of knowledge of HIV prevention, perceived HIV risk, and particularly HIV stigma among transport workers are still relevant in designing effective behavioural interventions to reduce risky sexual behaviours and adopt protective behaviours that reduce HIV transmission among transport workers. Studies conducted on the general populations among women and men in South Africa, Kenya, Bangladesh, Pakistan, and Chile point more to socio-demographic factors, such as; level of education, residence, and income levels as the most important predictors of level of knowledge of HIV prevention, perceived HIV risk, and HIV stigma.^12-16^ For instance, high levels of education and income was associated with higher levels of knowledge and lower levels of stigma in the general population.^12^ Similarly, high level of education was found to be associated with high HIV knowledge in studies conducted among truck drivers in Nigeria.^4,17^ On the other hand, male truck drivers and those with multiple concurrent sex partners were more likely to rate themselves as being at a higher risk of acquiring HIV.^18^

Nonetheless, there is still a dearth of literature on factors that influence HIV stigma perceptions among mobile workers such as transport workers. While some factors influencing perceived HIV risk and knowledge of HIV prevention are known in Uganda's context, gaps persist. Therefore, this study aimed to investigate the factors influencing the level of knowledge of HIV prevention, perceived HIV risk, and HIV stigma among transport workers in Mbarara City, located in southwestern Uganda. The findings of this study have implications for designing interventions aimed at reducing risky sexual behaviours and the subsequent HIV prevention among transport workers in Uganda and sub-Saharan Africa. Additionally, the prevention of HIV transmission among the most at-risk groups and their clients is paramount to achieving the global HIV elimination vision of zero new HIV infections, by 2025.^1^

## METHODS

### Study Design and Setting

The cross-sectional study was conducted in 4 slum areas found in 3 divisions of Mbarara city: Kiyanja, Ruti, Kakoba, and Kizungu. These areas are home to numerous transport workers namely; motor vehicle taxi drivers, motorcycle taxi riders, and stopovers for truck drivers, who work and make a living within and around these places daily. These communities are also resident to a large proportion of economically disadvantaged people, and sex workers. When compared to the national average of 5.4%, Mbarara district had the third highest level of HIV prevalence (13.1%) in Uganda in 2020.^19^

### Participants, Sample Size and Sampling Method

A sample of 424 transport workers was calculated using the Cochrane (1963) formula.^20^ When computing the sample size, the proportion of participants with one outcome of interest (e.g., HIV knowledge) was estimated to be 50% (*p = .5*), and the Z score and the allowable error were set at 1.96 and .05 respectively, generating a sample size of 385. The final sample size of 424 was obtained after adjusting for a 10% non-response rate. Participants were chosen using simple random sampling techniques (the lottery method with replacement).

Motorcycle taxi riders, truck and motor vehicle taxi drivers aged between 18 and 55 years, who had stayed, worked, or had multiple stopovers in Mbarara city for one year before the study period, and consented to participate in the study were recruited from their workplace, stopovers, or parks. Multiple stopovers were determined from participants' reports of having stopped (parked) and slept/lived in Mbarara city for more than 2 visits, regardless of the number of days.

### Data Collection

The survey was conducted for 10 weeks, between November 2021 and February 2022. Using simple random sampling techniques, participants were sampled using registers obtained from their association leaders. The leaders later introduced the research team to potential study participants, who were later contacted by the study team. Informed consent was obtained from the participants. Face to face interviews were conducted in either English or local (Runyakole/Rukiga) languages using a semi-structured questionnaire. The questionnaire was pretested with 10 motor vehicle taxi, truck drivers, and motorcycle taxi riders, in Ruharo Trading Centre, Mbarara City. The questionnaire was translated and back-translated from English to Runyakitara languages (Runyakole-Rukiga). Interviews were conducted in either language. The interviews were conducted at the participant's workplace, stop-overs, or parks. When a participant declined to participate in the study, two failed follow-up attempts were made before a replacement was sampled from the register provided by association leaders. The interviews took about 10 to 15 minutes and participants were given a token of appreciation in cash form upon completion of the interview, equivalent to 2 USD.

The study outcomes which included knowledge of HIV prevention, perceived HIV risk, and perceived HIV stigma were measured using different standardised semi-structured questionnaires. Knowledge of HIV prevention was assessed by asking 12 questions about HIV prevention using a questionnaire obtained from previous studies conducted in Cambodia and South Africa among female entertainment workers and the community.^21,22^ The answer options comprised “Yes”, “No” or “Don't Know”. A correct response for each item was awarded a score of 1 with a maximum score of 12. A binary dummy variable was created out of the total score which included poor (<6), and good/excellent knowledge (≥6) options. Perceived HIV risk was evaluated by asking participants to rate their level of agreement with statements (10 items) about perceived HIV risk obtained from the perceived risk questionnaire developed by Napper et.^11^ The Likert-like questions measure how one feels, understands, and thinks about the HIV risk. A maximum expected score of 43 was categorised as a low perceived HIV risk (<22.5), and a high perceived HIV risk (≥22.5). Perceived HIV stigma was assessed by asking participants to rate their level of agreement on the various 4-point Likert scale statements obtained from an 11-item questionnaire previously used to assess the perceived personal and community HIV stigma in South Africa.^23^ A maximum expected score of 44 was further characterised by low perceived HIV stigma (<22), and high perceived HIV stigma (≥22).

On the other hand, independent variables that were assessed in the study tools included sociodemographic characteristics and behavioural factors. Sociodemographic characteristics of the study participants that were assessed in this study include; gender, age, education, marital status, income level, length of stay in an area, occupation, and religion. Behavioural variables that influenced perceived HIV stigma, HIV risk, and knowledge of HIV prevention in previous studies were also assessed.^14-16^ These comprised the number of concurrent sex partners and prior HIV testing (knowing one's HIV status). During bivariate and multivariate analysis, outcome variables were also used as explanatory variables against one another as indicated in previous studies. For instance, HIV knowledge was used as an explanatory variable for perceived stigma. This is because lower HIV knowledge levels were previously found to be associated with higher HIV stigma perception.^21^ Whereas HIV knowledge and stigma explained perceived HIV risk.^15,16^

The Internal consistency of the knowledge of HIV prevention, perceived HIV risk, and HIV stigma subscales were assessed using the Cronbach alpha, which was found to be 0.707, 0.88, and 0.91, respectively.

### Data Analysis

Two data clerks cleaned and entered data in EpiData 3.1 software and later exported it to Statistical Package for the Social Sciences (SPSS), version 20. Dummy variables for continuous variables were created after computing total scores and running their reliability and normality analyses. The characteristics of the sample were described using descriptive statistics. The relationship between the outcomes and explanatory variables was established using Chi-square statistics. Binary logistic regression models for each of this study outcomes were computed for variables with *p<.1* and their adjusted odds ratios are reported at 95% confidence intervals.

### Ethical Consideration

Approval to conduct the study was obtained from the Mbarara University of Science and Technology research ethics committee (MUREC# 15/12-20). The study was conducted under the 1964 Declaration of Helsinki and its later amendments. Prior to the study, informed consent was obtained from all the study participants. Administrative clearance was obtained from the district health officer of the Mbarara district and the leaders of the transport workers.

## RESULTS

### Demographic Characteristics of the Study Participants

Table 1 summarises the demographic characteristics of the study participants. Out of 420 participants that were interviewed (99% response rate), 97.6% were males aged below 34 years (84.6%), with a median age of 28 years. Fifty-one percent (51%) had stayed temporarily, worked, or lived in the city for more than 5 years (a median of 6 years). The majority were Christians by faith (78.6%), single (41.2%), and had obtained an ordinary (39.5%) or primary-level certificate of education (38.5%). Nearly seventy percent (69.7%) were motorcycle taxi riders. Majority earned a monthly income of 400,001 to 500,000/= (~111 to 137 USD, 59.5%). Majority of the participants knew their current HIV serostatus (54.1%) and disclosed that they were seronegative (43.9%). However, most participants had multiple concurrent sex partners (52.4%).

**Table 1.** Demographic Characteristics of the Study Participants

Variable	n (%)
Gender	
Female	10 (2.5)
Male	400 (97.6)
Age in years	28 (18-48)*
>35	63 (15.4)
25-34	223 (54.4)
18-24	134 (30.2)
Length of stay in the area (years.)	6 (1-28)*
>5	209 (51.0)
<5	201 (49.0)
Marital status	
Single	169 (41.2)
Cohabiting	22 (5.4)
Married	154 (37.6)
Separated/divorced/widowed	65 (15.9)
Religion	
Catholic	118 (28.8)
Anglican	131 (32.0)
Moslem	84 (20.5)
Pentecost	73 (17.8)
Others	4 (1.0)
Education level	
Tertiary	11 (2.7)
Advanced level	56 (13.7)
Ordinary level	162 (39.5)
Primary	158 (38.5)
None	23 (5.6)
Occupation	
Truck driver/conductor	18 (4.4)
Motor vehicle driver/conductor	106 (25.9)
Motorcycle taxi rider (Boda Boda)	286 (69.7)
Monthly income (USD)	
>137	118 (28.8)
111-137	244 (59.5)
82-110	48 (11.7)
Sero-status (self-report)	
Unknown	188 (45.9)
Negative	180 (43.9)
Positive	42 (10.2)
Sex partners	2 (0-11)*
Multiple	215 (52.4)
Single	195 (47.6)

Note.

* Median (Minimum-Maximum), USD=United States dollars

### Knowledge about HIV Prevention

On the assessment of knowledge, results indicate that most of the transport workers had good knowledge of HIV prevention (69.3%), Table 2). Although the overall knowledge about HIV prevention is above average, gaps still exist. The majority of the participants had good knowledge or provided correct responses to items like: “Can men give HIV to women? (93.7%)”; “Can women give HIV to men?” (92.9%); “Is HIV caused by spirits/supernatural forces?” (82.9%); and “Can you get HIV by touching someone with HIV?” (70%). On the contrary, fewer participants correctly answered items such as: “Is HIV spread by kissing? (40.2%); “Does washing after sex help protect against HIV? (37.8%) and “Can a person get rid of HIV by having sex with a virgin? (35.6%).

**Table 2.** Knowledge about HIV Prevention

Items (correct options) Knowledge about HIV prevention Items	Yes n (%)	No/I don't know n (%)
Is HIV spread by kissing? (No)	245 (59.8)	165 (40.2) *
Does washing after sex help protect against HIV? (No)	255 (62.2)	155 (37.8) *
Can a person get HIV by sharing kitchens and bathrooms with someone with HIV? (No)	128 (31.2)	282 (68.8) *
Can men give HIV to women? (Yes)	384 (93.7) *	26 (6.3)
Can women give HIV to men? (Yes)	381 (92.9) *	29 (7.1)
Must a person have many different partners to get HIV? (No)	188 (45.1)	222 (54.1) *
Can you get HIV by touching someone with HIV? (No)	123 (30)	287 (70) *
Is HIV caused by spirits/supernatural forces? (No)	70 (17.1)	340 (82.9) *
Can a pregnant woman give HIV to her baby? (Yes)	268 (65.4) *	142 (34.6)
Can a person get rid of HIV by having sex with a virgin? (No)	254 (64.4)	146 (35.6) *
Is HIV the virus that causes AIDS? (Yes)	266 (69.8) *	124 (30.2)
Is there a cure for HIV? (No)	191 (46.6)	219 (53.4) *

Note. HIV, Human immune deficiency virus; AIDS acquired immune deficiency syndrome.

* = Good knowledge about HIV prevention.

### Perceived HIV risk

Most participants rated themselves as being at a high risk of acquiring HIV (75.4%, Table 3). Across items, many of the participants thought that “getting HIV is something they are concerned about (91%)”; at times “they worried about getting infected with HIV” (84.3%); thought that they were “likely to get infected with HIV?” (70.7%); “getting HIV is something they have thought about before” (66%), and they thought that “there is a chance, no matter how small, they could get HIV” (64.7%). However, fewer participants in this study found it difficult to “imagine themselves getting HIV” (56.4%) and thought that their chances of getting HIV were minimal (50.2%).

**Table 3.** Participants' Responses on the Perceived HIV Risk Scale

Perceived HIV risk Items	n (%)
Overall	
Low (L)	101 (24.6)
High (H)	309 (75.4)
What is your gut feeling about how likely you are to get infected with HIV?	
Extremely/very/somewhat unlikely (L)	120 (29.3)
Very/extremely likely (H)	290 (70.7)
I worry about getting infected with HIV	
None of the time (L)	64 (15.7)
Rarely/some/moderate/a lot/all of time (H)	344 (84.3)
Picturing self-getting HIV is something I find:	
Very hard/hard to do (L)	230 (56.4)
Very easy/easy to do (H)	178 (43.6)
Getting HIV is something I am…	
Not concerned (L)	37 (9)
Little/moderately/a lot extremely concerned (H)	373 (91)
I feel vulnerable to HIV infection	
Strongly Agree/Agree (H)	232 (56.9)
Strongly Disagree/Disagree (L)	176 (43.1)
There is a chance, no matter how small, I could get HIV	
Strongly Agree/Agree (H)	264 (64.7)
Strongly Disagree/Disagree (L)	144 (35.3)
I think my chances of getting infected with HIV are:	
Small/Almost zero/zero (L)	206 (50.2)
Moderate/large/very large (H)	204 (49.8)
Getting HIV is something I have	
Never/Rarely thought about (L)	139 (34)
Some/often of the time thought about (H)	270 (66)

Note. Two items were eliminated during reliability analyses, and these are not included in the table as indicated by Napper et al.^11^

L=low and H=high.

### Perceived HIV Stigma

When participants were asked to rate their level of perceived HIV stigma, The study found that most of the participants had a high level of perceived stigma (62%, Table 4), even on the subscales of interpersonal distancing (58.3%) and blame and Judgment (59%).

**Table 4.** Participant's Responses on the Perceived HIV Stigma Scale

Perceived HIV Stigma Item	Disagree (L) n (%)	Agree (H) n (%)
Overall	156 (38)	254 (62)
Blame and Judgment		
Overall	168 (41)	242 (59)
Getting HIV is a punishment for bad behavior	215 (52.7)	193 (47.3)
I feel afraid to be around people with HIV	260 (63.4)	150 (36.6)
People with HIV have only themselves to blame	217 (53.2)	191 (46.8)
If you have HIV, you must have done something wrong to deserve it	165 (40.9)	238 (59.1)
People with HIV should be ashamed of themselves	276 (67.8)	131 (32.2)
I feel uncomfortable around people with HIV	271 (66.9)	134 (33.1)
Interpersonal Distancing		
Overall	171 (41.7)	239 (58.3)
If I was on public or private transport, I would not like to sit next to someone with HIV	300 (73.2)	107 (26.3)
I think less of someone because they have HIV	165 (40.5)	242 (59.5)
I would not like someone with HIV to be living next door	293 (71.5)	117 (28.5)
I would not employ someone with HIV	305 (74.6)	104 (25.4)
I would not drink from a tap if a person with HIV had just drunk from it	170 (41.6)	239 (58.4)

Note. Agree=Strongly Agree/Agree; Disagree= Strongly disagree/disagree; L=low and H=high

Most participants thought: “Less of someone because they had HIV” (59.5%); “someone with HIV had done something wrong to deserve it (59.1%), and they would not drink from a tap if a person with HIV had just drunk from it (58.4%). Majority of the participants showed low levels of stigma or discriminatory attitudes on most of the items, for example; majority disagreed that: “I would not employ someone with HIV (74.6%)”; “If I was in public or private transport, I would not like to sit next to someone with HIV” (73.2%); “I would not like someone with HIV to be living next door” (71.5%); “People with HIV should be ashamed of themselves (67.8%), “I feel uncomfortable around people with HIV (66.9%)” and “I feel afraid to be around people with HIV” (63.4%).

### Factors Associated with Perceived HIV Stigma, HIV Risk, and Level of Knowledge of HIV Prevention

Using chi-square statistics, education level (*p* <.001, occupation (*p*=.015), age of the participants (*p* <.001), marital status (*p<.001*), number of concurrent sex partners (*p*=.019), level of income (*p*=.015), HIV status, and whether is known or not (*p <.001*), perceived HIV risk (*p <.001*) were statistically significantly associated with knowledge of HIV prevention, unlike gender (*p*=.15) and religion, (*p*=.292, Table 5).

**Table 5.** Factors Associated with the Level of Knowledge of HIV Prevention, Perceived HIV Stigma, and HIV Risk

Variable	Knowledge		Perceived HIV risk		Perceived Stigma	
	Poor (n%)	Good (n%)	Low (<) (n%)	High (>) (n%)	Low (<) (n%)	High (>) (n%)
Overall	126 (30.7)	284 (69.3)	101 (24.6)	309 (75.4)	156 (38.0)	254 (62.0)
Gender						
Male	125 (30.5) ns	225 (67.1)	99 (24.1) ns	301 (73.4)	149 (36.3)	51 (61.2)
Female	1 (0.2)	9 (2.2)	2 (0.5)	8 (2.0)	7 (1.7)	3 (0.7)
Occupation						
Motorcycle taxi rider (Boda Boda)	98 (23.9)	88 (45.9)	76 (18.5) ns	210 (51.2)	101 (24.6)	185 (45.1)
Motor vehicle Driver/conductor	27 (6.6)	79 (19.3)	22 (5.4)	84 (20.5)	42 (10.2)	64 (15.6)
Truck Driver/conductor	1 (0.2)	17 (4.1)	3 (0.7)	15 (3.7)	13 (3.2)	5 (1.2)
Age						
18-24	58 (14.1)*	66 (16.1)	51 (12.4)	73 (17.8)	36 (8.8)	88 (21.5)
25-34	53 (12.9)	170 (41.5)	42 (10.2)	181 (44.1)	88 (21.5)	135 (32.9)
≥35	15 (3.7)	48 (11.7)	8 (2.0)	55 (13.4)	32 (7.8)	31 (7.6)
Sex Partners						
Single	49 (12.0)*	146 (35.6)	44 (10.7) ns	151 (36.8)	82 (20.0) ns	113 (27.6)
Multiple	77 (18.8)	138 (33.7)	57 (13.9)	158 (38.5)	74 (18.0)	141 (34.4)
Marital status						
In relationship	31 (7.6)	145 (35.4)	76 (18.5)	158 (35.8)	68 (16.6)	166 (40.5)
Not in relationship	95 (23.2)	139 (33.9)	25 (6.1)	151 (36.8)	88 (21.5)	88 (21.5)
Religion						
Christians	103 (25.1) ns	219 (53.4)	84 (20.5) ns	238 (58.0)	115 (18.0) ns	207 (50.5)
Others	23 (5.6)	65 (15.9)	17 (4.1)	71 (17.3)	41 (10.0)	47 (11.5)
Income						
<500,000/= (below average)	100 (24.4)	192 (46.8)	75 (18.3) ns	217 (52.9)	105 (25.6) ns	187 (45.6)
≥500, 000/=	26 (6.3)	92 (22.4)	26 (6.3)	92 (22.4)	51 (12.4)	67 (16.3)
Education						
Primary and <	87 (21.2)	94 (22.9)	74 (18.0)	107 (26.1)	46 (11.2)	135 (32.9)
Secondary and >	39 (9.5)	190 (46.3)	27 (6.6)	202 (49.3)	110 (26.8)	119 (29.0)
HIV status						
Known	39 (9.5)	183 (35.4)	22 (5.4)	200 (48.8)	107 (26.1)	115 (28.0)
Unknown	87 (21.5)	101 (24.6)	79 (19.3)	109 (26.6)	49 (12.0)	139 (33.9)
Perceived stigma						
Low (Ref.)	-	-	16 (3.9)	140 (34.1)	-	-
High	-	-	85 (20.7)	169 (41.2)	-	-
Perceived Knowledge						
Poor	-	-	-	-	18 (4.4)	108 (26.3)
Good	-	-	-	-	138 (33.7)	146 (35.6)
Perceived HIV risk						
Low (Ref)	60 (14.6)	41 (10)	-	-	-	-
High	66 (16.1)	243 (59.3)	-	-	-	-

Note. ns=not statistically significantly associated; -were not compared with the outcome of interest due to lack of literature correlating the two variables.

Results indicate that education level (*p <.001*), HIV status (*p <.001*), age (*p <.001*), knowledge of HIV prevention (*p <.001*), perceived HIV stigma (*p* <.001), and marital status (*p <.001*), were statistically significantly associated with perceived HIV risk. Meanwhile, religion (*p*=.192), gender (*p*=.731), occupation (*p*=.358), number of concurrent sex partners (*p*=.354), and income levels (*p*=.437), were not associated with perceived HIV risk.

Gender (*p*=.035), marital status (*p <.001*), occupation (*p*=.007), age of the participants (*p*=.012), HIV status (*P<.001*), education level (*p <.001*), perceived HIV risk (*p <.001*), and knowledge of HIV prevention (*p* <.001), were statistically significantly associated with perceived HIV Stigma. The number of concurrent sex partners (*p*=.112), religion (*p*=.063), and income level (*p*=.170) were not associated with perceived HIV stigma.

### Predictors of Perceived HIV Stigma, Level of Knowledge about HIV Prevention, and Perceived HIV Risk

Factors associated with perceived stigma, knowledge of HIV prevention, and perceived HIV risk were entered into multivariate logistic regression models to evaluate their predictive and explanatory power. When the knowledge was compared with various factors, only the level of education (*p*=.002), knowing HIV status (*p*=.007), and perceived HIV risk (*p <.001*), statistically significantly predicted the level of knowledge of HIV prevention. Other variables in a model were not statistically significantly associated with the level of knowledge of HIV prevention including occupation (*p*=.122), age (*p*=.100), number of concurrent sex partners (*p*=.069), marital status (*p*=.11), and level of income (*p*=.057). Participants with high level of education (secondary and above) and high perceived HIV risk were 2.280 and 3.037 times more likely to have good level of knowledge of HIV prevention than those with a low level of education (primary or none) or low levels of perceived HIV risk. However, participants who did not know their HIV status were 0.472 times less likely to have good level of knowledge of HIV prevention.

When age, marital status, education level, HIV status, level of knowledge of HIV prevention, and perceived HIV stigma were compared with perceived HIV risk in logistic regression, only education level (*p*=.003), HIV status (*p <.001*), HIV knowledge (*p*=.003), and perceived stigma (*p*=.022), statistically significantly predicted perceived HIV risk. Age (*p*=.103), and marital status (*p*=.646), did not statistically significantly predict perceived HIV risk. The participants with high level of education (secondary and above) and good knowledge of HIV prevention were 1.340 and 2.380 times more likely to have high levels of perceived HIV risk than those with low level of education (primary or none) or poor knowledge of HIV prevention, respectively. However, participants who did not know their HIV status and those with a high level of perceived HIV stigma were 0.264 and 0.466 times less likely to have high level of perceived HIV risk than those who knew their HIV status or those with low levels of perceived HIV stigma.

Factors that statistically significantly predicted perceived HIV stigma included perceived HIV risk (*p*=.008), and knowledge of HIV prevention (*p <.001*, Table 6). Other variables in a model that were not statistically significantly associated with perceived HIV stigma included gender (*p*=.073), occupation (*p*=.255), age (*p*=.720), marital status (*p*=.065), religion (*p*=.383), education *(p*=.135), and knowing HIV status (*p*=.235). Participants with good knowledge of HIV prevention or a high perceived HIV risk were 0.296 and 0.411 times less likely to have a high level of HIV stigma than those with poor knowledge or low perceived HIV risk, respectively.

**Table 6.** Predictors of Perceived HIV Stigma, Level of Knowledge about HIV Prevention, and Perceived HIV Risk

Variable	Perceived Stigma AOR (95%CI)	Knowledge AOR (95%CI)	Perceived HIV risk AOR (95%CI)
Age			
18-24	1	1	1
25-34	1.272 (0.707-2.290)	1.491 (0.836-2.661)	1.818 (0.992-3.331)
≥35	1.188 (0.518-2.724)	0.708 (0.293-1.715)	2.332 (0.850-6.400)
Marital status			
In relationship	1	1	1
Not in relationship	0.618 (0.370 1.030)	1.626 (0.896-2.951)	0.856 (0.442-1.660)
Education			
Primary and <	1	1	1
Secondary and >	0.685 (0.418-1.125)	2.280 (1.355-3.836) *	1.340 (1.340-4.241) *
HIV status			
Known	1	1	1
Unknown	1.349 (0.823-2.209)	0.472 (0.274-0.813) *	0.264 (0.147-0.476) *
Occupation			
Motorcycle taxi rider (Boda Boda)	1	1	
Motor vehicle Driver/conductor	1.104 (0.650-1.878)	0.379 (0.089-1.604)	
Truck Driver/conductor	0.388 (0.114-1.317)	2.387 (0.185-30.856)	
Gender			
Male	1	-	-
Female	0.271 (0.065-1.130)	-	-
Sex Partners			
Single	-	1	-
Multiple	-	0.619 (0.369-1.038)	-
Religion			
Christians	1	-	-
Others	0.786 (0.458-1.350)	-	-
Income			
<500,000/= (below average)	-	1	-
≥500, 000/=	-	4.241 (0.958-18.768)	-
Perceived stigma			
Low (Ref.)	-	1	
High	-	0.466 (0.242-0.897) *	
Perceived Knowledge			
Poor	1	-	1
Good	0.296 (0.163-.536) *	-	2.380 (1.356-4.178) *
Perceived HIV risk			
Low (Ref)	1	1	-
High	0.411 (0.213-0.791) *	3.037 (1.735-5.315) *	-

Note. Bolded=significant

* Significant at 95% (p<0.05), and 99% (0.01) confidence interval, - Not included in the model.

## DISCUSSION

This study investigated the factors that predict HIV Knowledge, perceived HIV stigma, and HIV risk among transport workers in Mbarara City, southwestern Uganda. The findings of the survey suggest that although knowledge about HIV among most of the transport workers was above average or good (69%), it was still low when compared to results of other studies conducted in Nigeria, Namibia, Uganda, and South Africa which ranged from 37.5 to 97.4%.^4,17,18,24,25^ However, our findings are similar to results reported by a study conducted in eastern Nigeria among truck drivers which found knowledge about HIV spread and prevention to be low at about 51.3%.^26^ Multiple studies relate the main reason behind the difference in knowledge among transport workers across countries to the ease of accessing HIV-related information, most especially, through frequently targeted health outreaches and mass media campaigns.^27,28^ In addition, this study found gaps in knowledge about HIV prevention among transport workers that may warrant more sensitisation. For instance, numerous participants believed that one can get HIV through kissing and prevent HIV by washing after having unprotected sex or eliminate HIV by having sex with a virgin. This is similar to a study conducted in Nigeria which found that about 28.4% to 90.4% of the truck drivers had incorrect knowledge about HIV transmission with the majority holding various HIV misconceptions.^17^ Such misconceptions or beliefs about HIV transmission that are usually deep-rooted in culture may result in unprotected sex and thus need to be continuously redressed in health education sessions or campaigns.^29 30^

In this study, knowledge of HIV prevention was associated with occupation, marital status, age of the participants, number of concurrent sex partners, level of income, level of education, perceived HIV risk, knowing one's HIV status, unlike gender and religion. However, among drivers in Nigeria, marital status, age, and religion were found not to be significantly associated with knowledge about HIV transmission, except for the level of education.^4^

In multivariate analysis, participants who had acquired at least a high school level of education or above and those who had a high perceived HIV risk score were more likely to possess good level of knowledge of HIV prevention compared with those with primary or no formal education at all or those with low perceived HIV scores. This partly concurs with studies conducted among transport workers in Nigeria, which found that those drivers who had high level of education were more likely to have high level of knowledge of HIV transmission.^4,17^ On the contrary, participants who did know their HIV serostatus were less likely to have good knowledge about HIV prevention compared to those who knew their HIV status, as shown in a study conducted in Zambia among long-distance workers. ^31^ This means that continuous HIV testing has influence on increasing HIV knowledge among transport workers, thus this strategy needs to be prioritised and encouraged among the different social groups.

The study also found that the majority of the participants rated themselves as being at high risk of acquiring HIV, similar to results reported by a study conducted in Nigeria (87.6%) among bus drivers, but much lower than an earlier study that reported it at 12.9% and 34% among Nigerian and Namibian truck drivers respectively.^4,18,32^ The differences in awareness and knowledge about HIV prevention which has been found to influence perceived HIV risk may explain the variations in findings across and within countries. Future researchers may explore the reasons why despite a high perceived HIV risk, numerous participants in this study felt that their chances of acquiring HIV were low or they did not imagine themselves having HIV, since such perceptions perpetuate risky sexual and HIV transmission behaviours.^4^

The age of the participants, marital status, education level, HIV status, knowledge of HIV prevention, and perceived HIV stigma were statistically significantly associated with perceived HIV risk in this study, unlike religion, gender, occupation, income levels, and the number of sex partners. On the contrary, among transport workers from Namibia, occupation, and gender were found to be significantly associated with perceived HIV risk, whereas age and marital status were not.^18^ Nonetheless, in logistic regression, participants with a high level of education (secondary and above) and good knowledge of HIV prevention compared with their counterparts were more likely to have a high level of perceived HIV risk. This was partly consistent with results of a study conducted in Nigeria where a high level of education was found to be associated with a high perceived risk of acquiring HIV.^17^ A high education level and HIV knowledge may convey a better understanding of HIV, thus making transport workers perceive a high risk of HIV infection. For instance, in Nigeria, highly educated truck drivers knew well the transmission routes of HIV which changed their perceptions about the risk of acquiring HIV.^17^ In addition, participants who did not know their HIV status and those with a high perceived HIV stigma were less likely to have a high perceived HIV risk when compared with their counterparts. Possibly, those who knew their HIV status may have undertaken an HIV test because they perceived the risk of acquiring HIV to be high. On the other hand, individuals who possess high stigmatising attitudes toward people living with HIV are more likely to have a high perception of HIV risk contrary to our study findings.^15^

The perceived stigma which means “a feeling that those without the condition perceive people with the condition negatively” or “perceptions about how stigmatised groups are treated in a particular context”, remains a global problem.^29,33^ Perceived Stigma has been found to fuel the HIV epidemic through reducing the utilisation of HIV testing services, non-disclosure of HIV status, delay in initiation, and adherence to ART.^9,29,33^ Unlike an earlier study in Uganda about motorcycle taxi riders that found a low level of HIV stigma (4.6%),^24^ this study found that most of the participants had a high level of perceived HIV stigma (62%). Similarly, a study conducted in Nigeria among long-distance drivers found it to be as high as 86.7%.^17^ In addition, among this study participants, there was an overlap of the social or interpersonal distancing stigmatizing attitudes, that are a precursor for HIV-related discrimination with those related to blame or judgment. However, further research is needed to find the key drivers and facilitators of these stigmatising attitudes among transport workers before they are redressed through a wide range of individual, group (interpersonal), and community interventions.^29^

This study also found that gender, occupation, age of the participants, marital status, education level, HIV status, perceived HIV risk, and knowledge of HIV prevention were significantly associated with perceived HIV Stigma. However, only perceived HIV risk and knowledge of HIV prevention predicted perceived HIV stigma in multivariate analysis. Particularly, participants with good knowledge about HIV prevention or a high perceived HIV risk were less likely to have a high level of HIV stigma. In the general population, a high level of stigma has been associated with lower education levels, lack of literacy skills, lack of knowledge, and cultural beliefs about HIV.^21^ In conclusion, this study seems to suggest that the level of education and awareness of one's HIV status positively influences HIV knowledge and perceived risk. Whereas HIV-related stigma was in turn negatively influenced by the transport workers' HIV knowledge and perceived risk.

### Limitations of the Study

This study has limitations that may influence its findings. Namely, some participants either declined or did not complete the interview because of a busy work schedule. Findings from such participants may have enriched the study.

The cross-sectional study design does not allow for causal attributions.

Last, the administration of the questionnaire by interviewers carried the risk of social desirability bias. Nevertheless, the study utilised a large sample size and had a high respondent rate (99%), replaced participants who dropped out of the study, and reassured participants about the benefits of the study and/or why they needed to provide appropriate answers during the interviews.

## CONCLUSIONS

The study found a large proportion of participants with good knowledge about HIV prevention, a high HIV perceived risk and stigma. The findings suggest the need to continue addressing HIV stigma by the various stakeholders. Probably, stakeholders need to start by understanding and further investigating the facilitators and drivers of HIV stigma among mobile workers and tailor out strategies to them. Accordingly, the stigmatising attitudes about HIV among these groups may reflect the societal, cultural, and social beliefs of the communities that these transport workers come from, thus wider policies beyond their networks are needed. Thus, multifaceted approaches at the individual, group (interpersonal), and community levels are critical to reducing HIV stigma among this study group. In addition, this study seems to suggest that the level of education and awareness of one's HIV status positively influences HIV knowledge and perceived risk. Whereas HIV-related stigma was in turn negatively influenced by the transport workers' HIV knowledge and perceived risk. This calls for continuous health education programs on HIV and encouraging HIV testing among transport workers.

## References

[1] UNAIDS. EN DANGER. 2022; Accessed December 23, 2022. https://www.unaids.org

[2] UNAIDS. Creating HIV prevention cascades. 2021; Accessed December 23, 2022. https://www.unaids.org

[3] Malta M, Bastos F, Pereira-Koller E, Cunha M, Marques C, Strathdee SA. A qualitative assessment of long distance truck drivers' vulnerability to HIV/AIDS in Itajai, southern Brazil. AIDS care. 2006;18(5):489–496. doi: 10.1080/09540120500235241 16777641

[4] Awosan K, Ibrahim MO, Arisegi S, Erhiano E. Knowledge of HIV/AIDS, risk perception, sexual lifestyle and condom use among drivers in Sokoto, Nigeria. Journal of Infectious Diseases and Immunity. 2014;6(3):19–25. doi: 10.5897/JIDI2013.0129

[5] Lalla-Edward ST, Ncube S, Matthew P, Hankins CA, Venter WDF, Gomez GB. Uptake of health services among truck drivers in South Africa: analysis of routine data from nine roadside wellness centres. BMC Health Serv Res. Sep 13 2017;17(1):649. doi: 10.1186/s12913-017-2595-3 28903727 PMC5598062

[6] Delany-Moretlwe S, Bello B, Kinross P, et al. HIV prevalence and risk in long-distance truck drivers in South Africa: a national cross-sectional survey. Int J STD AIDS. May 2014;25(6):428–38. doi: 10.1177/0956462413512803 24352131

[7] Mathias T, Emmanuel OO, Kakwezi MR, John M. Prevalence and predisposing factors of human immunodeficiency virus infection among the boda-boda riders in Mbarara municipality-Uganda. 2020; Accessed December 23, 2022. https://nru.uncst.go.ug

[8] UNAIDS. Country factsheets Uganda. 2019; Accessed January 23, 2023. https://www.uac.go.ug/

[9] Subedi B, Timilsina BD, Tamrakar N. Perceived stigma among people living with HIV/AIDS in Pokhara, Nepal. HIV AIDS (Auckl). 2019;11:93–103. doi: 10.2147/HIV.S181231 31118826 PMC6510232

[10] Caliari JS, Teles SA, Reis RK, Gir E. Factors related to the perceived stigmatization of people living with HIV. Rev Esc Enferm USP. Oct 9 2017;51:e03248. doi: 10.1590/S1980-220X2016046703248 29019527

[11] Napper LE, Fisher DG, Reynolds GL. Development of the perceived risk of HIV scale. AIDS Behav. May 2012;16(4):1075–83. doi: 10.1007/s10461-011-0003-2 21785873 PMC3338003

[12] Iqbal S, Maqsood S, Zafar A, Zakar R, Zakar MZ, Fischer F. Determinants of overall knowledge of and attitudes towards HIV/AIDS transmission among ever-married women in Pakistan: evidence from the Demographic and Health Survey 2012–13. BMC Public Health. 2019;19(1):1–14. doi: 10.1186/s12889-019-7124-3 31226969 PMC6588857

[13] Yaya S, Bishwajit G, Danhoundo G, Seydou I. Extent of Knowledge about HIV and Its Determinants among Men in Bangladesh. Front Public Health. 2016;4:246. doi: 10.3389/fpubh.2016.00246 27857939 PMC5093132

[14] Ochako R, Ulwodi D, Njagi P, Kimetu S, Onyango A. Trends and determinants of Comprehensive HIV and AIDS knowledge among urban young women in Kenya. AIDS research and therapy. 2011;8(1):1–8. doi: 10.1186/1742-6405-8-11 21375746 PMC3060857

[15] Cianelli R, Villlegas N, De Oliveira G, et al. Predictors of HIV enacted stigma among Chilean women. Journal of clinical nursing. 2015;24(17-18):2392–2401. doi: 10.1111/jocn.12792 25693422 PMC4544676

[16] Maughan-Brown B, Venkataramani AS. Accuracy and determinants of perceived HIV risk among young women in South Africa. BMC public health. 2018;18(1):1–9. doi: 10.1186/s12889-017-4593-0 PMC5520344 28732496

[17] Ijeoma A, Ejikeme A, Theodora O, Chika O. Knowledge, attitude, willingness of HIV counseling and testing and factors associated with it, among long distant drivers in Enugu, Nigeria: an opportunity in reduction of HIV prevalence. Afr Health Sci. Dec 2018;18(4):1088–1097. doi: 10.4314/ahs.v18i4.30 30766575 PMC6354848

[18] Kiderlen TR, Conteh M, Roll S, Seeling S, Weinmann S. Cross-sectional study assessing HIV-related knowledge, attitudes and behavior in the Namibian truck transport sector: Readjusting HIV prevention programs in the workplace. J Infect Public Health. Jul-Aug 2015;8(4):346–54. doi: 10.1016/j.jiph.2015.02.001 25805432

[19] Betunga B, Atuhaire P, Nakasiita C, et al. Factors influencing the use of multiple HIV prevention services among transport workers in a city in southwestern Uganda. PLOS Global Public Health. 2023;3(3):e0001350. doi: 10.1371/journal.pgph.0001350 36962980 PMC10021771

[20] Israel GD. Determining sample size. 1992; Accessed November 23, 2022. https://www.tarleton.edu

[21] Kalichman SC, Simbayi LC. HIV testing attitudes, AIDS stigma, and voluntary HIV counselling and testing in a black township in Cape Town, South Africa. Sexually transmitted infections. 2003;79(6):442–447. doi: 10.1136/sti.79.6.442 14663117 PMC1744787

[22] Yi S, Tuot S, Chhoun P, et al. Sexual behaviors, HIV knowledge, HIV testing attitudes and recent HIV testing among female entertainment workers in Cambodia: A cross-sectional study. PLoS One. 2018;13(7):e0198095. doi: 10.1371/journal.pone.0198095 29965968 PMC6028079

[23] Visser MJ, Makin JD, Vandormael A, Sikkema KJ, Forsyth BW. HIV/AIDS stigma in a South African community. AIDS Care. Feb 2009;21(2):197–206. doi: 10.1080/09540120801932157 19229689 PMC4238924

[25] Disan M. Factors Influencing the Uptake of Selected HIV Prevention Methods among Boda-Boda Riders in Fort Portal Municipality Kabarole District Uganda. Global Scientific Journal. 2022;10(3). Accessed May 23, 2024. http://www.globalscientificjournal.com/

[25] Ssekankya V, Githaiga SK, Aleko T, et al. Factors Influencing Utilization of HIV Testing Services among Boda-Boda Riders in Kabarole District, Southwestern Uganda: A Cross-Sectional Study. Biomed Res Int. 2021;2021:8877402. doi: 10.1155/2021/8877402 33869636 PMC8035009

[26] Ibe Sally N, Nwoke E, Emerole C, Onyeocha F, Nwawume I, Nwaokoro JC. Knowledge, attitude and perception of road transport workers to HIV/AIDS, STIs and condom use in southeast, Nigeria. Technology. 2014;1(2):50–54. Accessed November 23, 2022. https://www.semanticscholar.org

[27] Kelvin EA, George G, Mwai E, et al. Offering Self-administered Oral HIV Testing as a Choice to Truck Drivers in Kenya: Predictors of Uptake and Need for Guidance While Self-testing. AIDS Behav. Feb 2018;22(2):580–592. doi: 10.1007/s10461-017-1783-9 28540563 PMC5818565

[28] Kelvin EA, George G, Mwai E, et al. Offering self-administered oral HIV testing to truck drivers in Kenya to increase testing: a randomized controlled trial. AIDS Care. Jan 2018;30(1):47–55. doi: 10.1080/09540121.2017.1360997 28826229 PMC5901679

[29] UNAIDS. Global AIDS Monitoring 2022. 2021; Accessed December 23, 2022. https://www.unaids.org

[30] Darteh EKM, Abraham SA, Seidu A-A, Chattu VK, Yaya S. Knowledge and determinants of women's knowledge on vertical transmission of HIV and AIDS in South Africa. AIDS research and therapy. 2021;18(1):1–9. doi: 10.1186/s12981-021-00367-7 34266455 PMC8281453

[31] Mutale LS, Chola M, Chongwe G, et al. Uptake of HIV testing and its associated factors among long-distance truck drivers in Zambia, 2015. Journal of Interventional Epidemiology and Public Health. 2018;1. doi: 10.37432/jieph.2018.1.1.10

[32] Ekanem E, Afolabi B, Nuga A, Adebajo S. Sexual behaviour, HIV-related knowledge and condom use by intra-city commercial bus drivers and motor park attendants in Lagos, Nigeria. African Journal of Reproductive Health. 2005:78–87. Accessed December 23, 2022. https://www.ajrh.info 16104657

[33] Mukerji R, Turan JM. Exploring Manifestations of TB-Related Stigma Experienced by Women in Kolkata, India. Ann Glob Health. Nov 5 2018;84(4):727–735. doi: 10.29024/aogh.238310.9204/aogh.2383 30779523 PMC6748300

